# Systematically profiling the expression of eIF3 subunits in glioma reveals the expression of eIF3i has prognostic value in IDH-mutant lower grade glioma

**DOI:** 10.1186/s12935-019-0867-1

**Published:** 2019-06-04

**Authors:** Rui-Chao Chai, Ning Wang, Yu-Zhou Chang, Ke-Nan Zhang, Jing-Jun Li, Jun-Jie Niu, Fan Wu, Yu-Qing Liu, Yong-Zhi Wang

**Affiliations:** 1 Department of Molecular Neuropathology, Beijing Neurosurgical Institute, Beijing Tiantan Hospital, Capital Medical University, No. 119 Nan Si Huan Xi Road, Fengtai District, Beijing, 100160 China; 2 Department of Clinical Laboratory, Beijing Chao-Yang Hospital, Capital Medical University, Beijing, 100020 China; 3 Department of Neurosurgery, Beijing Tiantan Hospital, Capital Medical University, No. 119 Nan Si Huan Xi Road, Fengtai District, Beijing, 100160 China; 4 China National Clinical Research Center for Neurological Diseases, Beijing Tiantan Hospital, Capital Medical University, Beijing, 100160 China; 5 Xiang Fen Centers for Disease Control and Prevention, Xiangfen, 041500 Shanxi China; 6 Chinese Glioma Genome Atlas Network (CGGA), Beijing, China

**Keywords:** eIF3, Glioma, 1p/19q codeletion, Prognosis, Biomarker

## Abstract

**Background:**

Abnormal expression of the eukaryotic initiation factor 3 (eIF3) subunits plays critical roles in tumorigenesis and progression, and also has potential prognostic value in cancers. However, the expression and clinical implications of eIF3 subunits in glioma remain unknown.

**Methods:**

Expression data of eIF3 for patients with gliomas were obtained from the Chinese Glioma Genome Atlas (CGGA) (*n* = 272) and The Cancer Genome Atlas (TCGA) (*n* = 595). Cox regression, the receiver operating characteristic (ROC) curves and Kaplan–Meier analysis were used to study the prognostic value. Gene oncology (GO) and gene set enrichment analysis (GSEA) were utilized for functional prediction.

**Results:**

In both the CGGA and TCGA datasets, the expression levels of eIF3d, eIF3e, eIF3f, eIF3h and eIF3l highly were associated with the IDH mutant status of gliomas. The expression of eIF3b, eIF3i, eIF3k and eIF3m was increased with the tumor grade, and was associated with poorer overall survival [All Hazard ratio (HR) > 1 and P < 0.05]. By contrast, the expression of eIF3a and eIF3l was decreased in higher grade gliomas and was associated with better overall survival (Both HR < 1 and P < 0.05). Importantly, the expression of eIF3i (located on chromosome 1p) and eIF3k (Located on chromosome 19q) were the two highest risk factors in both the CGGA [eIF3i HR = 2.068 (1.425–3.000); eIF3k HR = 1.737 (1.166–2.588)] and TCGA [eIF3i HR = 1.841 (1.642–2.064); eIF3k HR = 1.521 (1.340–1.726)] databases. Among eIF3i, eIF3k alone or in combination, the expression of eIF3i was the more robust in stratifying the survival of glioma in various pathological subgroups. The expression of eIF3i was an independent prognostic factor in IDH-mutant lower grade glioma (LGG) and could also predict the 1p/19q codeletion status of IDH-mutant LGG. Finally, GO and GSEA analysis showed that the elevated expression of eIF3i was significantly correlated with the biological processes of cell proliferation, mRNA processing, translation, T cell receptor signaling, NF-κB signaling and others.

**Conclusions:**

Our study reveals the expression alterations during glioma progression, and highlights the prognostic value of eIF3i in IDH-mutant LGG.

**Electronic supplementary material:**

The online version of this article (10.1186/s12935-019-0867-1) contains supplementary material, which is available to authorized users.

## Background

Diffused gliomas are the most common malignant primary brain tumors, which accounts for more than 75% of adult malignant primary brain tumors [1, 2]. Currently, the clinical outcomes for patients with gliomas are still generally poor, and the median overall survival (OS) of patients with glioblastoma-IDH-wildtype (GBM-IDH-wt), WHO grade IV is only approximately 15 months even after comprehensive treatment with surgery, radiotherapy, and chemotherapy [1, 3–6]. Patients with lower-grade glioma (LGGs, WHO grade II and III) have relatively better prognosis, but most of these tumors relapse as therapy-resistant higher-grade gliomas over time [7, 8]. Along with the discovery of the significant clinical implications of isocitrate dehydrogenase (IDH) mutation and 1p/19q codeletion in stratifying the gliomas, these two molecular markers have been combined with the traditional histopathological examination to form an integrated diagnosis for diffuse gliomas in the 2016 Classification of Tumors of the Central Nervous System [5, 9, 10]. The IDH-mutant and 1p/19q codeletion LGG has the best prognosis among all the subgroups of the integrated diagnosis [11–15]. Compared with gliomas in other subgroups, the decreased expression of genes located on chromosomes 1p and 19q is a significant characteristic for the IDH-mutant and 1p/19q codeletion LGG [16], indicating some of these genes may affect the prognosis of gliomas.

Increased protein synthesis has been shown to be required for the oncogenesis of human cancers [17]. The eukaryotic initiation factor (eIF) complexes are responsible for regulating the initiation step of protein synthesis, and the eIF3, eIF4 and eIF5 complexes have been reported to be associated with cancers [18, 19]. Among these eIF complexes, the eIF3 complex contains 13 subunits, which are essential during the initiation of protein synthesis for their roles in maintaining 40S ribosomal subunit in a dissociated state and forming 43S pre-initiation complex [19, 20]. Importantly, eIF3 has been shown to be involved in the process of carcinogenesis through regulating the translations of some specific mRNAs, including cell-proliferation mRNAs [20]. Moreover, it has been shown that over-expression of eIF3 subunits eIF3a, eIF3b, eIF3c, eIF3i, eIF3h and eIF3m could promote the malignant transformation of various cancers [21–23]. Therefore, the aberrant expression of eIF3 subunits has been thought of important candidate biomarkers for cancer prognosis and target-therapy [19]. Additionally, eIF3 subunits eIF3i and eIF3k are located on the chromosomes 1p and 19q, respectively. All of these suggest that the expression of eIF3 subunits may have potential clinical significances in gliomas.

To elucidate the clinical significance of eIF3 subunits expression in gliomas, we systemically analyzed the expression of thirteen eIF3 subunits in 867 gliomas with RNA sequencing and molecular pathological data from the Chinese Glioma Genome Atlas (CGGA) (n = 272) and The Cancer Genome Atlas (TCGA) (n = 595) datasets. We revealed the expression profile of all eIF3 subunits in gliomas with different clinicopathological features and also expounded the prognostic value of each eIF3 subunit in gliomas. Based on this, we further investigated the prognostic value of eIF3i (located on chromosome 1p), eIF3k (located on chromosome 19q), and the combination of eIF3i and eIF3k in gliomas with specific molecular features. Finally, our study highlighted the prognostic value and potential biological association of eIF3i in IDH-mutant LGG.

## Materials and methods

### Patients and data collection

In this study, we collected 272 glioma patients from the Chinese Glioma Genome Atlas (CGGA, http://www.CGGA.org.cn) with transcriptome sequencing data and molecular pathological data. The clinical information for these patients has been collected, and the Overall survival (OS) was calculated from the diagnosing date to the death date or last follow-up date. This study was approved by the Beijing Tiantan Hospital institutional review board (IRB), and handwriting informed consent was acquired from each patient. There are 595 gliomas patients, whose transcriptome sequencing data and clinical information could be available in The Cancer Genome Atlas (TCGA) RNAseq database (http://cancergenome.nih.gov), were also included in this study. The clinical information for all these 867 patients is summarized in Additional file 1: Table S1.

### eIF3 expression analysis in datasets

The expression levels of all eIF3 subunits were shown with the heatmap by pheatmap packages in the R (https://www.r-project.org/, v3.4.3). The expression levels of eIF3 subunits were compared in gliomas with subgroups of WHO 2016 classification or IDH status both the CGGA and TCGA database by the GraphPad Prism 7 (GraphPad Software Inc., La Jolla, CA).

In addition, the GEPIA software (a website analyzing the RNA sequencing expression data from the TCGA and GTEx projects) was utilized to compare the expression of eIF3i in glioma and normal brain tissue (http://gepia.cancer-pku.cn/detail.php).

### Bioinformatics analysis

The Pearson correlation analysis was performed by R language to calculate the correlation between eIF3i and other genes in CGGA and TCGA RNAseq datasets, respectively. Gene Ontology (GO) and Kyoto Encyclopedia of Genes and Genomes (KEGG) pathway enrichment analyses were performed with Database for Annotation, Visualization, and Integrated Discovery (DAVID) (http://david.abcc.ncifcrf.gov/home.jsp) to functionally annotate genes that significantly (Pearson r > 0.5 or < − 0.5, and the adjusted *P* value Bonferroni correction < 0.05) correlated to the eIF3i.

### Statistical analysis

Student’s t-test and one-way ANOVA test were used to test the significance of differences for eIF3 subunits between gliomas with different clinicopathological features. The Cox regression analysis was employed to evaluate the correlations among eIF3 subunits and the combination of eIF3i and eIF3k in gliomas. The receiver operating characteristic (ROC) curves were used to study the predictive efficiencies of eIF3i, eIF3k, and the combination of eIF3i and eIF3k on the survival status of patients with glioma.

Kaplan–Meier survival analysis and the log-rank test were used to assess the statistical significance between the low-expression and high-expression groups stratified by expression of eIF3I, and the median expression of eIF3I was used as cut-off value.

Univariate and multivariate Cox regression analysis including gender, age at diagnosis, WHO grade, and 1p/19q codeletion status were used to assess prognostic value of EIF3I expression in IDH-mutant LGGs. The ROC curve was used to study the predictive efficiency of EIF3I expression on 1p/19q codeletion status in IDH-mutant LGGs.

All statistical analyses were performed with R (https://www.r-project.org/, v3.4.3), SPSS 16.0 (SPSS Inc., Chicago, IL), and GraphPad Prism 7 (GraphPad Software Inc., La Jolla, CA). The P value of 0.05 was taken as the significant threshold in all tests.

## Results

### The expression of eIF3 subunits in gliomas with different pathological features

**Fig. 1:**
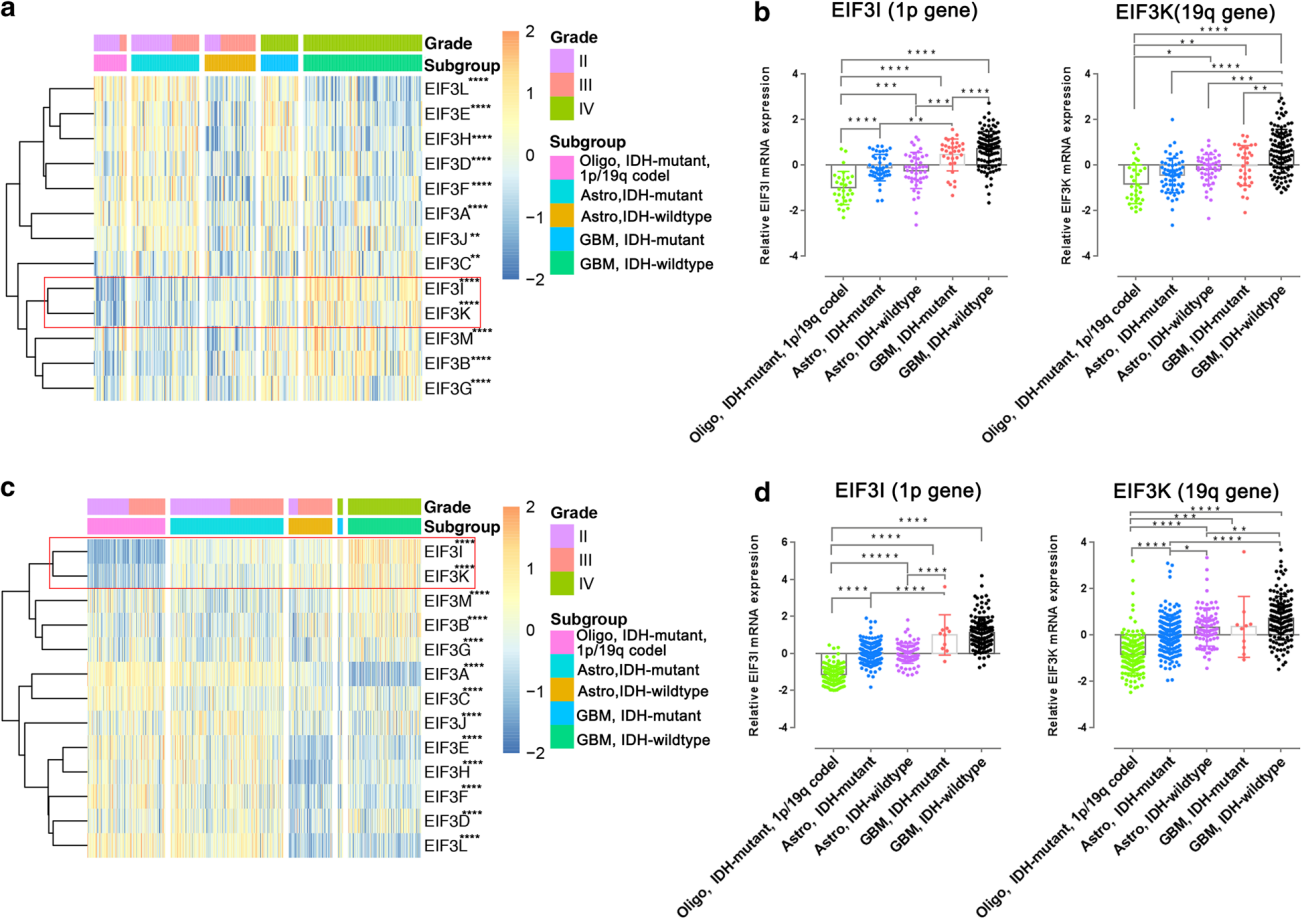
The expression of eIF3 subunits in gliomas with different pathological features. Heatmaps showing the expression levels of thirteen eIF3 subunits in gliomas with different pathological features according to WHO 2016 integrated diagnosis from the CGGA (**a**) and TCGA (**c**) datasets. Statistic data showing the expression levels of eIF3i (locate on chromosome 1p) and eIF3k (located on chromosome 19q) in gliomas with different pathological features according to WHO 2016 integrated diagnosis from the CGGA (**b**) and TCGA (**d**) datasets. *P < 0.05, **P < 0.01, *** P < 0.001 and ****P < 0.0001

To get an overview for the expression of eIF3 subunits in glioma, we constructed a gene expression heatmap with all thirteen eIF3 subunits in gliomas with different pathological features according to the WHO 2016 integrated diagnosis (Fig. 1a, c). The results showed that all eIF3 subunits were significantly differentially expressed among different subgroups of gliomas in both CGGA (Fig. 1a, all P < 0.01) and TCGA datasets (Fig. 1c, all P < 0.0001). Among these eIF3 subunits, the expression of eIF3a was negatively correlated with the WHO grade of gliomas, especially in the TCGA dataset (Additional file 1: Fig. S1A, C). The expression of eIF3d, eIF3e, eIF3f, eIF3h and eIF3l were significantly different between IDH-mutant and IDH-wildtype gliomas (Additional file 1: Fig. S1B, D), and this differential expression was not associated with the gender (Additional file 1: Fig. S2). Importantly, we noticed that the expression of both eIF3i (located on chromosome 1p) and eIF3k (Located on chromosome 19q) were positively correlated with the malignancy of gliomas (Fig. 1b, d). Both eIF3i and eIF3k had the highest expression in IDH-wildtype GBM, but had the lowest expression in IDH-mutant, 1p/19q codeletion, oligodendrogliomas.

### The prognostic value of eIF3 subunits in gliomas

We next sought to investigate the prognostic values of eIF3 subunits in gliomas. We performed a univariate Cox regression analysis on the expression levels of these eIF3 subunits in both the CGGA and TCGA dataset (Table 1). The results indicated that the expression levels of eIF3a, eIF3b, eIF3i, eIF3k, eIF3l and eIF3m were significantly (P < 0.05) correlated to the OS of glioma patients in both datasets. Among these eIF3 subunits, the eIF3a and eIF3l were protective subunits with hazard ratio (HR) less than 1, while eIF3b, eIF3i, eIF3k and eIF3m were risky subunits with HR larger than 1, and eIF3i (located on chromosome 1p) and eIF3k (Located on chromosome 19q) were the two most risky subunits in both CGGA [eIF3i HR = 2.068 (1.425–3.000); eIF3k HR = 1.737 (1.166–2.588)] and TCGA [eIF3i HR = 1.841 (1.642–2.064); eIF3k HR = 1.521 (1.340–1.726)] datasets.

**Table 1:** The correlation between eIF3 subunits and the OS of patients with gliomas

	CGGA dataset				TCGA dataset			
	P-value	HR	Confidence interval		P-value	HR	Confidence interval	
			Low 95%	High 95%			Low 95%	High 95%
*eIF3a*	*1.397E−03*	*0.598*	0.436	0.820	*0.000E+00*	*0.489*	0.437	0.548
*eIF3b*	*1.546E−02*	*1.449*	1.073	1.957	*1.099E−05*	*1.395*	1.203	1.618
eIF3c	5.471E*−*01	1.094	0.817	1.465	3.258E*−*06	0.737	0.648	0.838
eIF3d	3.072E*−*01	0.835	0.591	1.180	6.551E*−*03	0.803	0.685	0.940
eIF3e	2.538E*−*01	0.844	0.630	1.130	4.034E*−*10	0.596	0.507	0.701
eIF3f	1.929E*−*01	0.787	0.550	1.128	5.040E*−*08	0.645	0.550	0.755
eIF3g	6.377E*−*01	1.102	0.736	1.649	6.549E*−*01	0.965	0.824	1.130
eIF3h	5.883E*−*02	0.728	0.524	1.012	3.061E*−*06	0.682	0.581	0.801
*eIF3i*	*1.306E−04*	*2.068*	1.425	3.000	*0.000E+00*	*1.841*	1.642	2.064
eIF3j	9.257E*−*01	0.981	0.659	1.460	4.766E*−*04	0.773	0.669	0.893
*eIF3k*	*6.640E−03*	*1.737*	1.166	2.588	*8.229E−11*	*1.521*	1.340	1.726
*eIF3l*	*9.946E−03*	*0.704*	0.539	0.919	*0.000E+00*	*0.437*	0.374	0.511
*eIF3m*	*3.926E−02*	*1.421*	1.017	1.984	*1.369E−03*	*1.262*	1.095	1.456

Italic values indicate the statistically significant difference (P < 0.05)

Considering the clinical implication of 1p/19q codeletion in gliomas, we also compared the prognosis ability of eIF3i, eIF3k alone or in combination in stratified gliomas from CGGA dataset. The combined expression of eIF3i and eIF3k = eIF3i expression*1.048 + eIF3k expression * 0.797, and the 1.048 and 0.797 were the log2 of HR values for eIF3i and eIF3k in COX regression, respectively.

The LGG included IDH-wildtype LGG (IDH-wildtype astrocytoma) and IDH-mutant LGG, and the IDH-mutant LGG could be further stratified as 1p/19q codeletion IDH-mutant oligodendroglioma and IDH-mutant astrocytoma (1p/19q non-codeletion). In the patients with LGG (Table 2), the expression of eIF3i and combined expression of eIF3i and eIF3k had significant prognostic value in total LGG and each stratified LGG, including IDH-wildtype astrocytoma, all IDH-mutant LGG and IDH-mutant astrocytoma, while the expression of eIF3k only had prognostic value in total LGG and total IDH-mutant LGG. We were unable to study the prognostic value of these factors in 1p/19q codeleted IDH-mutant oligodendroglioma due to the low ratio (< 10%, n = 2) of deceased patients. The GBM included IDH-wildtype GBM and IDH-mutant GBM (Table 3). Only the expression of eIF3i had prognostic value in total GBM and IDH-mutant GBM.

**Table 2:** The prognostic value of eIF3i, eIF3k and the combination of eIF3i and eIF3k in LGG (n = 134 in CGGA dataset)

eIF3 subunit	LGG				Astro, IDH-wildtype				All LGG, IDH-mutant				Astro, IDH-mutant			
	HR	Confident interval		P-value	HR	Confident interval		P-value	HR	Confident interval		P-value	HR	Confident interval		P-value
		Low 95%	High 95%			Low 95%	High 95%			Low 95%	High 95%			Low 95%	High 95%	
*eIF3i*	*3.59*	*2.30*	*5.62*	*2.12E−08*	*2.90*	*1.44*	*5.83*	*2.87E−03*	*4.00*	*2.15*	*7.44*	*1.24E−05*	*4.41*	*2.02*	*9.61*	*1.94E−04*
*eIF3k*	*2.13*	*1.39*	*3.27*	*5.61E−04*	1.24	0.64	2.39	5.27E*−*01	*2.36*	*1.31*	*4.24*	*4.03E−03*	1.78	0.92	3.47	8.86E*−*02
*eIF3i and eIF3k*	*2.22*	*1.65*	*2.98*	*1.44E−07*	*1.82*	*1.14*	*2.91*	*1.16E−02*	*2.34*	*1.56*	*3.52*	*3.80E−05*	*2.12*	*1.33*	*3.36*	*1.51E−03*

Italic values indicate the statistically significant difference (P < 0.05)

**Table 3:** The prognostic value of EIF3I, EIF3K and the combination of EIF3I and EIF3K in GBM (n = 138 in CGGA dataset)

EIF3 subunit	GBM				GBM, IDH-wildtype				GBM, IDH-mutant			
	HR	Confident interval		P-value	HR	Confident interval		P-value	HR	Confident interval		P-value
		Low 95%	High 95%			Low 95%	High 95%			Low 95%	High 95%	
*EIF3I*	*1.56*	*1.16*	*2.09*	*3.51E−03*	1.31	0.96	1.80	9.05E*−*02	*5.74*	*1.57*	*20.92*	*8.12E−03*
EIF3K	1.02	0.83	1.26	8.51E*−*01	0.87	0.68	1.12	2.88E*−*01	1.27	0.74	2.17	3.90E*−*01
EIF3I and EIF3K	1.16	0.99	1.37	6.33E*−*02	1.05	0.87	1.25	6.34E*−*01	1.62	1.00	2.64	5.10E*−*02

Italic values indicate the statistically significant difference (P < 0.05)

**Fig. 2:**
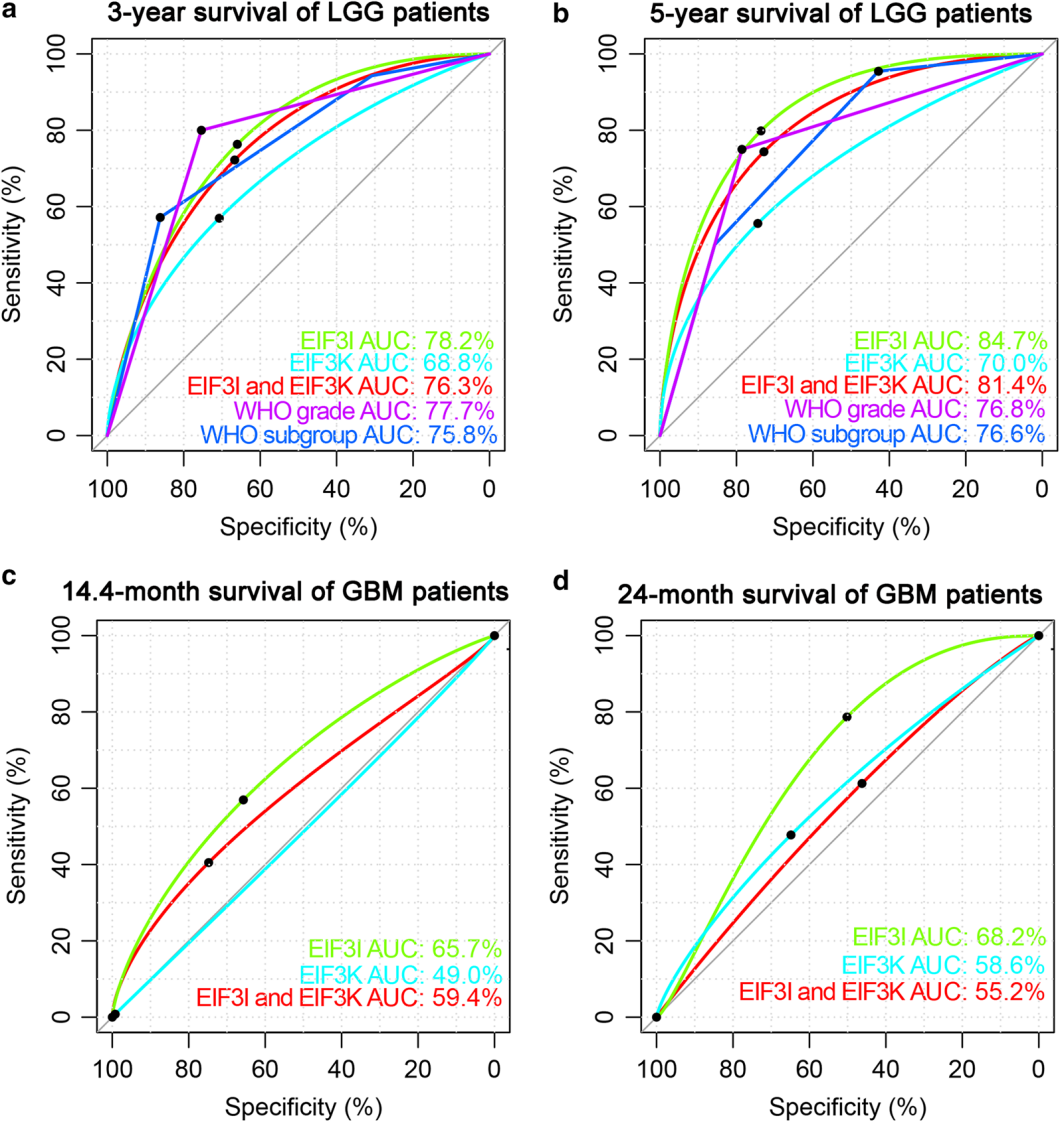
The predict efficiencies of eIF3i and eIF3k in gliomas. ROC curves showed the predictive efficiencies of eIF3i, eIF3k, combination of eIF3i and eIF3k, WHO grade and subgroups of WHO 2016 integrated diagnosis (Oligodendroglioma, IDH-mutant, 1p/19q codeletion; Astrocytoma, IDH-mutant; Astrocytoma, IDH-wildtype) on 3-year (**a**) and 5-year survival (**b**) in LGG of CGGA dataset. ROC curves showed the predictive efficiencies of eIF3i, eIF3k and combination of eIF3i and eIF3k on 14.4-month (**c**) and 24-month survival (**d**) in GBM of CGGA dataset

We also compared the prognostic efficiencies of eIF3i expression, eIF3k expression and combined expression of eIF3i and eIF3k through ROC curves (Fig. 2). The results showed that the expression of eIF3i had the superior predictive efficiency compared with eIF3k expression, combined expression of eIF3i and eIF3k, WHO grade and subgroups of WHO 2016 for predicting survival of patients with 1p/19q non-codeletion glioma in the CGGA dataset (Fig. 2a, b). In the LGG, Areas under the curve (AUCs) of eIF3i, eIF3k, combination of eIF3i and eIF3k, WHO grade and subgroups of WHO 2016 were 78.2%, 68.8%, 76.3%, 77.7% and 75.8% respectively for 3 year’s survival; 84.7%, 70.0%, 81.4%, 76.8% and 76.6% respectively for 5 year’s survival. In the GBM, AUCs of eIF3i, eIF3k and combination of eIF3i and eIF3k were 65.7%, 49.0% and 59.4% respectively for 14.4 month’s survival; 68.2%, 58.6% and 55.2% respectively for 24 year’s survival.

### Expression of eIF3i is increased in higher grade gliomas

**Fig. 3:**
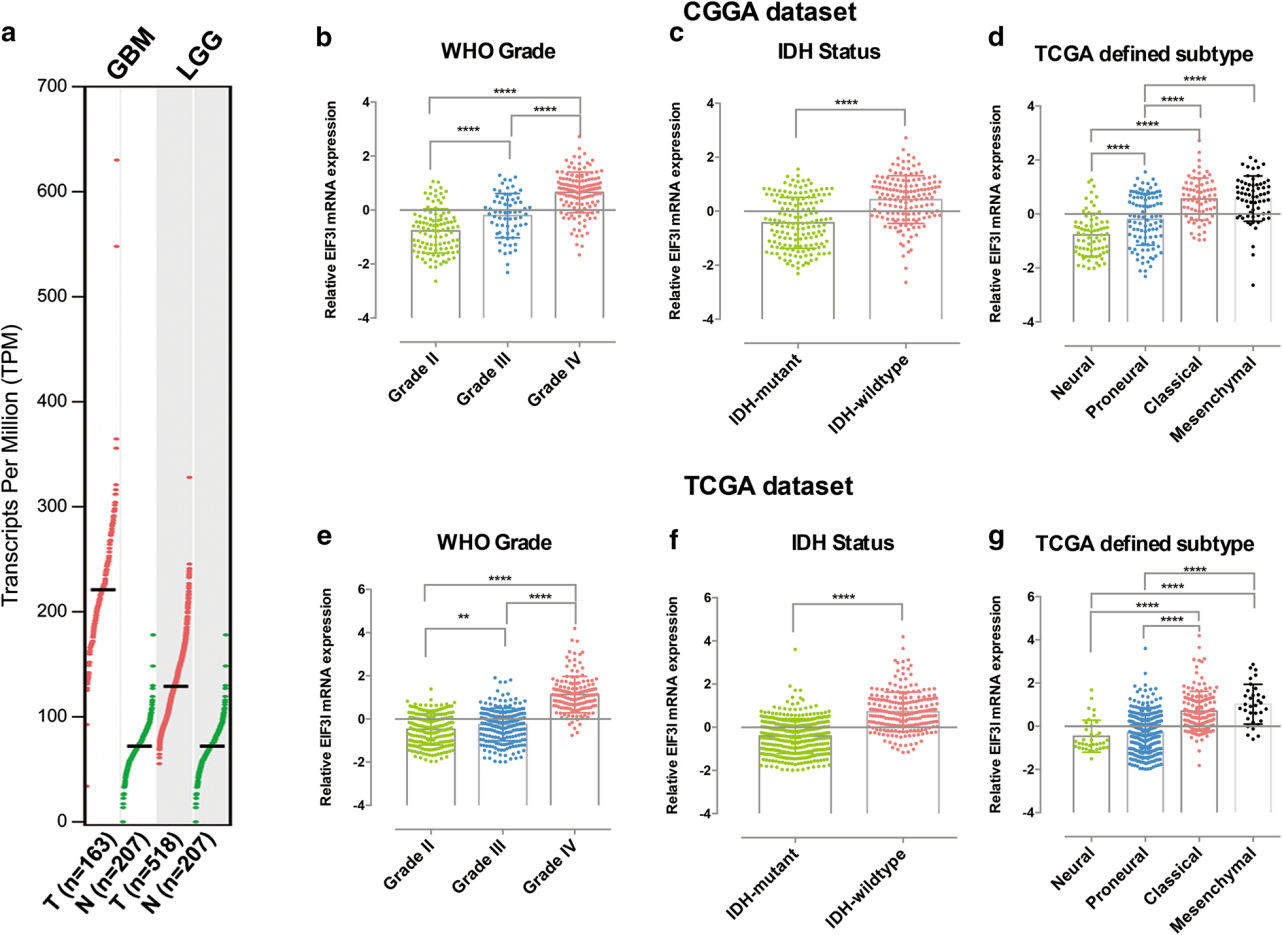
The expression of eIF3i in normal brain tissue and gliomas with different clinicopathological features. **a** The expression profiles of eIF3i mRNA in each GBM, LGG, normal brain tissues were represented. Quantification data shows the expression levels of eIF3i in gliomas from the CGGA (**b**–**d**) and TCGA (**e**–**g**) datasets stratified by the WHO grade (**b**, **e**), IDH status (**c**, **f**) TCGA defined subtype (**d**, **g**). **P < 0.01 and ****P < 0.0001

Based on above findings, we focused our investigation on the eIF3i in the grade and prognosis of gliomas. To get an overview of eIF3i expression in gliomas and normal brain tissues, we analyzed the expression of eIF3i expression in the GEPIA database. The expression profiles of eIF3i in GBM, LGG and normal brain tissue were represented (Fig. 3a). The expression of eIF3i in GBM was higher than that in both LGG and normal brain tissues, and the expression of eIF3i in the LGG is also higher than that in normal brain tissues (Fig. 3a). To investigate the association between the EIF3i expression and the clinicopathological features of gliomas, we further compared the expression of eIF3i in gliomas stratified by WHO grade and IDH status. In the CGGA dataset, the expression of eIF3i was significantly increased along with the increasing WHO grades (both P < 0.0001, Fig. 3b), and the expression of eIF3i was also significantly (P < 0.0001) increased in IDH-wildtype gliomas (Fig. 3c). The similar results could also be observed in the TCGA dataset (Fig. 2e, f).

To insight the molecular relevance of eIF3i expression in gliomas, we also investigated the expression of eIF3i in gliomas with different molecular subtypes defined by the TCGA [24]. The results showed that the eIF3i had higher expression in the Classical and Mesenchymal gliomas in both CGGA (Fig. 2d) and TCGA (Fig. 2g) datasets.

### The expression of eIF3i could predict the 1p/19q codeletion status in IDH-mutant LGGs

As eIF3i locates on the chromosome 1p, we also investigated the association between the expression of eIF3i and the 1p/19q codeletion status of IDH-mutant LGG (Additional file 1: Fig. S3). We observed that the expression of eIF3i was significantly decreased in 1p/19q codeletion and IDH-mutant LGG in both the CGGA (P < 0.0001, Additional file 1: Fig. S3A) and TCGA (P < 0.0001, Additional file 1: Fig. S3C) datasets. In addition, the ROC analysis indicated that the expression of eIF3i could also predict the 1p/19q codeletion statuses of IDH-mutant LGG in both the CGGA (AUC = 83.1%, Additional file 1: Fig. S3B) and TCGA (AUC = 95.9%, Additional file 1: Fig. S3D) datasets.

### The expression of eIF3i is an independent prognostic indicator in IDH-mutant LGGs

To address the potential clinical implications of eIF3i in gliomas, we also investigated the prognostic value of eIF3i expression in total glioma, LGG and GBM through Kaplan–Meier curves (Additional file 1: Fig. S4). Patients with gliomas were divided into high-expression and low-expression groups by the median expression level of eIF3i in total gliomas, LGGs and GBMs, respectively. We found that patients in low-expression groups had significantly longer OS than those in high-expression groups when total gliomas in CGGA (P < 0.0001, Additional file 1: Fig. S4A), LGGs in CGGA (P < 0.0001, Additional file 1: Fig. S4B), total gliomas in TCGA (P < 0.0001, Additional file 1: Fig. S4D) and LGGs in TCGA (P = 0.01, Additional file 1: Fig. S4E) were analyzed, respectively. In patients with GBM, though the OS of patients in the low-expression group was longer (P = 0.092, Additional file 1: Fig. S4C) than that of high-expression group in the CGGA dataset, the median expression level of eIF3i cannot stratify the OS (P = 0.305, Additional file 1: Fig. S4F) of patients in TCGA dataset.

**Fig. 4:**
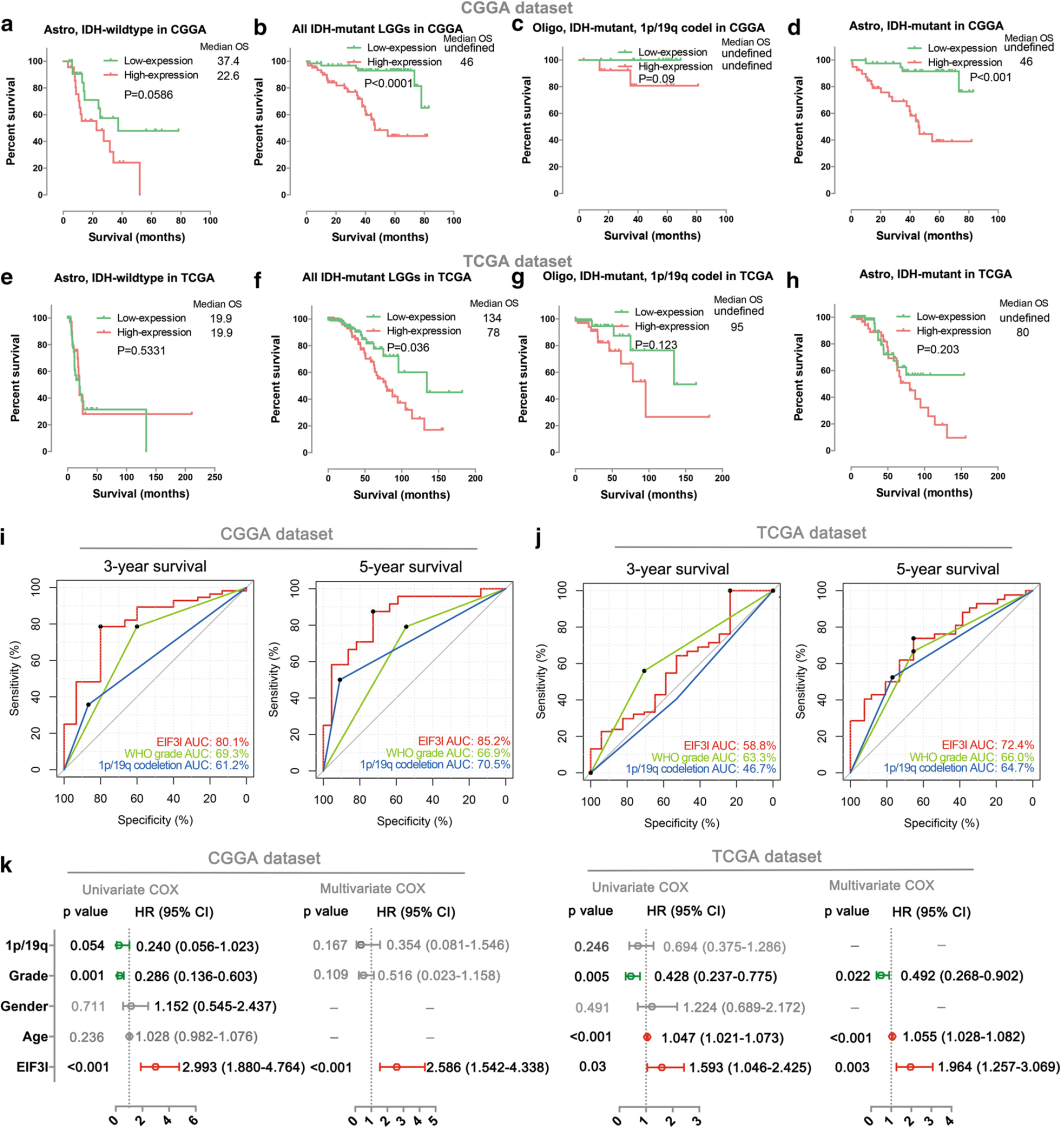
Prognostic value of eIF3i expression in stratified LGG, especially in IDH-mutant LGG. **a**–**h** Kaplan–Meier overall survival curves for patients stratified by the respective median expression of eIF3i in the CGGA (**a**–**d**) and TCGA (**e**–**h**) datasets with astrocytoma, IDH-wildtype (**a**, **e**), all IDH-mutant LGGs (**b**, **f**), oligodendroglioma, IDH-mutant, 1p/19q codel (**c**, **g**) and astrocytoma, IDH-mutant (**d**, **h**), respectively. **i**, **j** ROC curves showed the predictive efficiencies of eIF3i, WHO grade (II and III) and 1p/19q codeletion status on 3-year and 5-year survival in TCGA (I) and CGGA (J) datasets. **k** Univariate and multivariate Cox regression analyses of the association between clinicopathological factors (including the eIF3i expression) and overall survival of patients in the CGGA and TCGA datasets

In addition, we also studied the prognostic value of the eIF3i expression in stratified LGGs and GBMs. In LGGs, though patients with IDH-wildtype astrocytoma in low-expression group had longer (P = 0.059) OS than that of high-expression group in the CGGA database (Fig. 4a), the expression of eIF3i cannot stratify the OS of patients in TCGA dataset (P = 0.533, Fig. 4e). However, eIF3i expression could stratify the OS of patients with IDH-mutant LGGs in both CGGA (P < 0.0001, Fig. 4b) and TCGA (P = 0.036, Fig. 4f) dataset. Furthermore, we also noticed that there was a tendency that the patients with low-expression of eIF3i had longer OS than patients with high-expression of eIF3i in both 1p/19q co-deleted, IDH-mutant oligodendroglioma (P = 0.09 for CGGA and P = 0.123 for TCGA, Fig. 4 C and G) and 1p/19q non-codeletion IDH-mutant astrocytoma (P < 0.001 for CGGA and P = 0.203 for TCGA, Fig. 4d, h).

In the GBMs, though there were only a small number of patients with IDH-mutant GBM included in this study, the median expression of eIF3i also showed the potential to stratify the OS of these patients in both CGGA (P = 0.0602, n = 33) and TCGA (P = 0.3532, n = 10) datasets, while the median expression of eIF3i cannot stratify the OS of patients with IDH-wildtype GBM in both CGGA and TCGA dataset (Additional file 1: Fig. S5).

Then we further investigated the prognostic value of eIF3i in IDH-mutant LGGs. The ROC curve analysis showed that the eIF3i expression had a superior predictive efficiency compared with WHO grade and 1p/19q codeletion status for predicting the 3-year and 5-year survival of patients with IDH-mutant LGG in CGGA dataset (Fig. 4i). AUCs of eIF3i, WHO grade and 1p/19q codeletion status were 80.1%, 69.3% and 61.2% respectively for 3 year’s survival; 85.2%, 66.9% and 70.5% respectively for 5 year’s survival. The similar results could also be validated in the TCGA database (Fig. 4j), AUCs of eIF3i, WHO grade and 1p/19q codeletion status were 58.8%, 63.3% and 46.7% respectively for 3 year’s survival; 72.4%, 66.0% and 64.7% respectively for 5 year’s survival.

Finally, we also performed univariate and multivariate Cox regression analyses to determine whether the expression level of eIF3i was an independent prognostic indicator for IDH-mutant LGGs in both the CGGA and TCGA datasets (Fig. 4k). To our surprise, the 1p/19q codeletion is not significant (P = 0.246) correlated to the OS of IDH-mutant LGGs in the TCGA dataset. However, compared with other clinicopathological features, including 1p/19q co-deletion status, WHO Grade, gender and age, the expression level of eIF3i was significantly correlated with the OS of IDH-mutant LGGs by multivariate Cox analysis in both the CGGA (P < 0.001) and TCGA (P = 0.003) datasets. This analysis confirmed that the expression level of eIF3i could independently predict prognosis in patients with IDH-mutant LGGs.

### Functional annotation of eIF3i in IDH-mutant LGGs

**Fig. 5:**
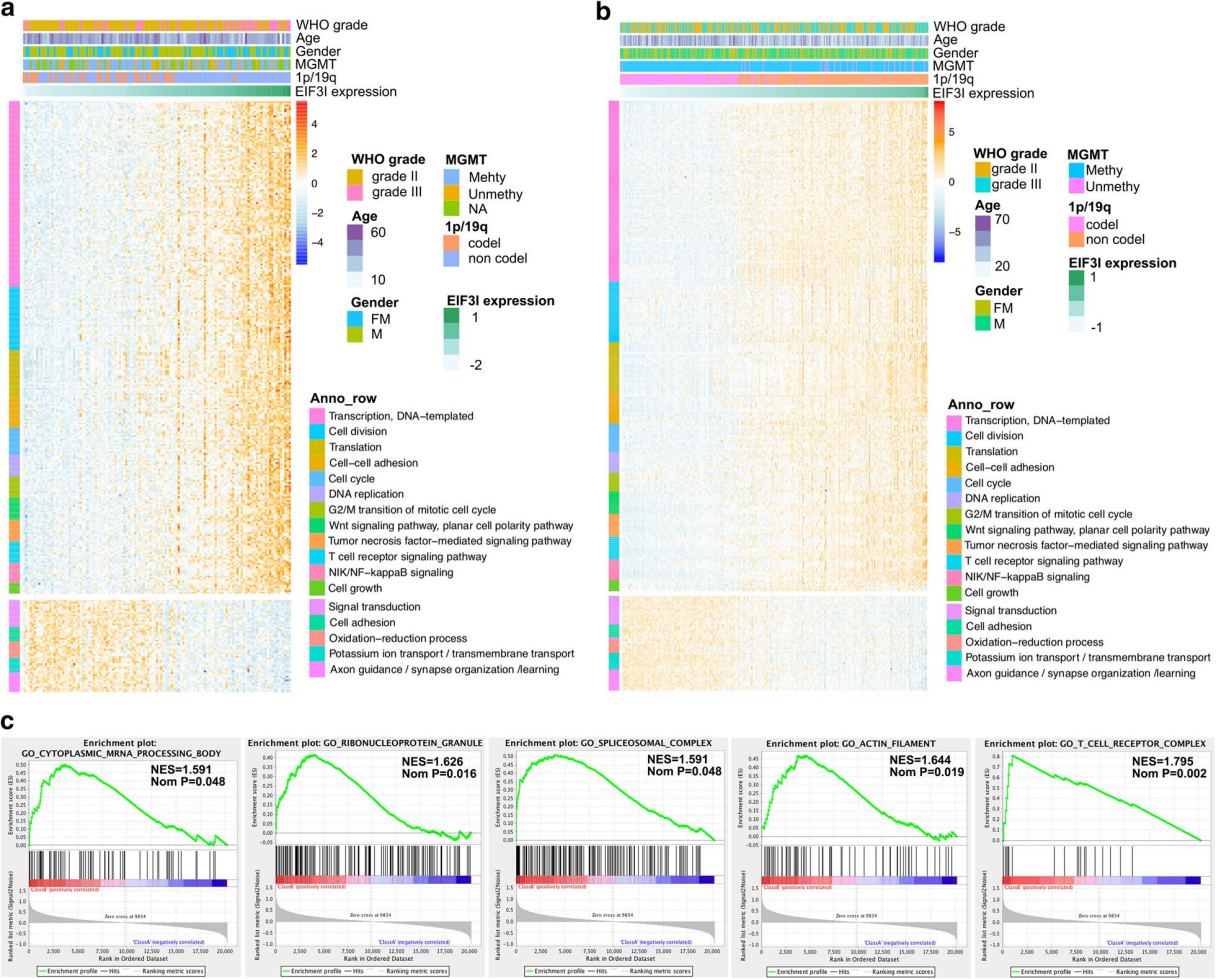
Functional annotation for genes significantly correlated to eIF3i expression in IDH-mutant LGG. Biological processes of genes that correlated to eIF3i expression in CGGA (**a**) and TCGA (**b**) datasets. **c** GSEA revealed enriched GO terms positively correlated to EIF3I expression in CGGA dataset

To explore the potential functions that were associated with eIF3i expression in LGGs, we identified genes that significantly positively (r > 0.5, normalized P < 0.05) or negatively (r < − 0.5, normalized P < 0.05) correlated with the eIF3i expression, and then annotated their functions using GO terms of BP in CGGA (Fig. 5a) and TCGA (Fig. 5b) databases. Genes which were positively correlated with eIF3i expression were primarily involved in tumor progression, including “transcription”, “cell division”, “translation”, “cell–cell adhesion”, “cell cycle”, “DNA-replication”, “tumor necrosis factor-mediated signaling pathway”, “T cell receptor signaling pathway”, “NF-kappa B signaling” and “Cell growth”. In contrast, the genes that were negatively correlated with the expression of eIF3i were closely related to the normal neuronal functions, such as “signal transduction”, “cell adhesion”, “Oxidation–reduction process”, “Potassium ion transport/transmembrane transport” and “Axon guidance/synapse organization/learning”.

Moreover, GSEA analysis was also carried out to validate the functions that were correlated with eIF3i expression (Fig. 5c), indicating that “cytoplasmic mRNA processing body”, “ribonucleoprotein granule”, “Spliceosomal complex”, “actin filament” and “T cell receptor complex” were highly positively correlated to the expression of eIF3i (All normalized P < 0.05).

## Discussion

In this study, we systemically revealed the expression profiles of all thirteen eIF3 subunits among gliomas with different pathological features, and then we also analyzed their prognostic value in gliomas. We noticed that the elevated expression of eIF3i (located on chromosome 1p) and eIF3k (located on chromosome 19q) were significantly correlated with the increased malignancy of glioma. We also compared the prognosis ability of eIF3i, eIF3k alone or in combination, and the results showed that the expression of eIF3i was the more robust in stratifying the survival of glioma in various pathological subgroups. Furthermore, the expression of eIF3i was also an independent prognostic factor in IDH-mutant LGG. Finally, we observed that the expression of eIF3i was significantly associated with the biological progresses of cell proliferation, mRNA processing, translation, T cell receptor signaling, NF-κB signaling and others. Molecular classification of malignancies of gliomas is important to establish the diagnosis and to develop the target-therapy for gliomas with similar histological or pathological features [3, 25, 26]. Although specific individual subunits of eIF3 have been revealed to be up-regulated or down-regulated in numerous human tumors [19, 20, 22, 27–29], our study further reveals the expression profile and clinical implications of eIF3 subunits in gliomas.

It has been reported that eIF3i can promote colon oncogenesis by regulating COX-2 protein synthesis and beta-catenin activation [1]. Increased expression of eIF3i has been used as a theranostic marker for using Akt specific inhibitors in human hepatocellular carcinoma [30]. Here, we addressed the prognostic value of eIF3i in gliomas, especially in IDH-mutant gliomas through analyzing large samples. The expression of eIF3i was not only an independent prognostic indicator in IDH-mutant LGG but also had prognostic value in IDH-mutant GBM. Moreover, we also identified that eIF3i expression was significantly correlated with the biological processes of cell division, cell growth and translation. This is consistent with the reports that elevated eIF3i could promote cell proliferation and angiogenesis in tumorigenesis through up-regulated VEGFA translation [23, 29].

IDH mutant and 1p/19q codeletion LGGs are less invasive and have better prognosis than other gliomas [31–34]. However, the underlying mechanism is still unclear. Here, we noticed that the expression of eIF3i could predict the 1p/19q codeletion status in IDH-mutant LGGs, and the eIF3i expression was significantly decreased in IDH-mutant and 1p/19q codeletion LGGs. Considering the vital roles of eIF3i in tumor progression mentioned above, the decreased expression of eIF3i may be one of the mechanisms underlying the better prognosis of IDH-mutant and 1p/19q codeletion LGGs.

As a bioinformatics study, the study only revealed the functions that were possibly associated with eIF3i expression and the definite functions of eIF3i in glioma are still needed to be further confirmed. However, our study not only revealed the clinical implications and potential functions of eIF3i in gliomas but also revealed the expression changes of all eIF3 subunits during glioma progression. Considering that abnormal expression of these factors have been reported to be involved in various pathological conditions, including cancers [3, 19–21, 27, 28, 35], our findings supplied basic information for future investigating the roles of these eIF3 subunits in gliomas.

## Conclusion

In summary, we profiled the expression of eIF3 subunits among gliomas with different pathological features and revealed the correlations of each eIF3 subunits with OS of glioma patients. Based on these, we systemically investigated the prognostic values of eIF3i (located on chromosome 1p) and eIF3k (located on chromosome 19q) in stratified glioma and identified that the expression of eIF3i was closely correlated with the OS of patients in serval stratified glioma subgroups. The elevated expression of eIF3i was also correlated with pathological features of gliomas and was an independent prognostic factor eIF3i in IDH-mutant LGG. Bioinformatic analysis predicted that elevated expression eIF3i was involved cell proliferation, Inflammation and T cell related signaling pathways in IDH-mutant LGG.

## Additional file

**Additional file 1.** Additional figures and tables.

## Data Availability

All transcriptional data used in this study were available from CGGA database (http://www.cgga.org.cn) and TCGA database (http://cancergenome.nih.gov). Other information is available through contacting the corresponding authors.

## Abbreviations

eIF3: eukaryotic initiation factor 3
CGGA: the Chinese Glioma Genome Atlas
TCGA: The Cancer Genome Atlas
ROC: the receiver operating characteristic
GO: gene oncology
GSEA: gene set enrichment analysis
HR: hazard ratio
LGG: lower grade glioma
GBM: glioblastoma
IDH: isocitrate dehydrogenase
DAVID: Database for Annotation, Visualization, and Integrated Discovery
