# Supplementary material for: Systematically profiling the expression of eIF3 subunits in glioma reveals the expression of eIF3i has prognostic value in IDH-mutant lower grade glioma
Source: Cancer Cell Int. 2019 Jun 4;19:155. doi: 10.1186/s12935-019-0867-1 (PMC6549376; doi:10.1186/s12935-019-0867-1)
Supplement: Supplementary file 1 — Additional file 1. Additional figures and tables. [file 12935_2019_867_MOESM1_ESM.pdf]

Table S1 Clinicopathological features of patients used in this study

	CGGA dataset		TCGA dataset	
	Number	Percentage	Number	Percentage
**Total**	272	100.00%	595	100.00%
**Age**	8-81 (43)		14-89 (47)	
<median	128	47.06%	292	49.08%
⩾ median	144	52.94%	303	50.92%
**Gender**				
Female	102	37.50%	248	41.68%
Male	170	62.50%	343	57.65%
**Grade**				0.00%
II	73	26.84%	211	35.46%
III	61	22.43%	235	39.50%
IV	138	50.74%	149	25.04%
**IDH**				
Mutation	122	44.85%	373	62.69%
Wildtype	150	55.15%	222	37.31%
**1p19q**				
Codel	29	10.66%	148	24.87%
Non-codel	243	89.34%	447	75.13%
**Subgroups of WHO 2016**				
Oligo, IDH-mutant 1p/19q codel	29	10.66%	148	24.87%
Astro, IDH-mutant	60	22.06%	215	36.13%
Astro, IDH-wildtype	45	16.54%	83	13.95%
GBM IDH-mutant	33	12.13%	10	1.68%
GBM IDH-wildtype	105	38.60%	139	23.36%
**TCGA subtype**				
Neural	59	21.69%	36	6.05%
Proneural	81	29.78%	378	63.53%
Classical	68	25.00%	148	24.87%
Mesenchymal	64	23.53%	33	5.55%

Figure S1. The expression of eIF3a, eIF3d/e/f/h/l in gliomas.

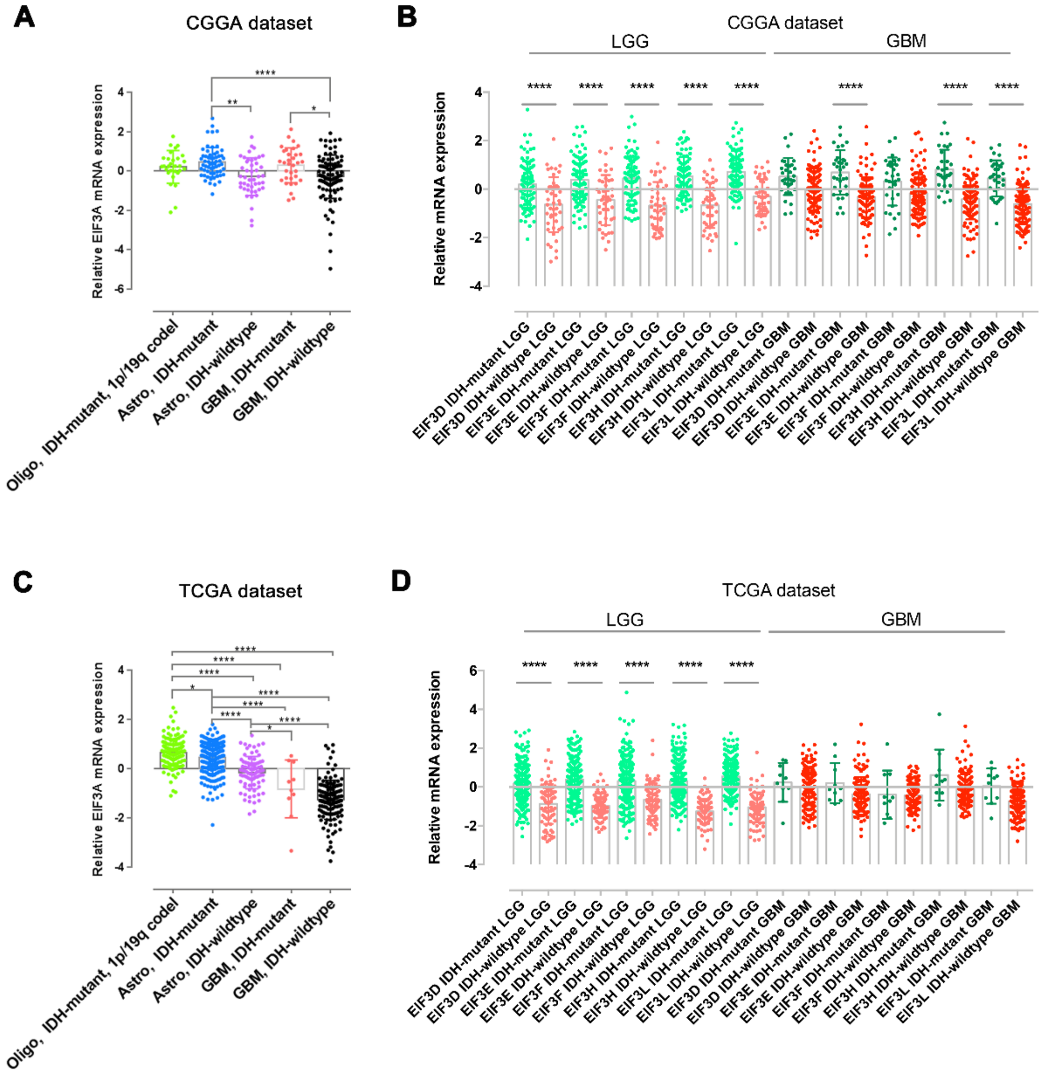

(A and C) Statistic data showing the expression levels of eIF3a in gliomas with different pathological features according to WHO 2016 integrated diagnosis from the CGGA (A) and TCGA (C) datasets. **(B-D)** Quantification data shows the expression levels of eIF3d/e/f/h/l in gliomas from the CGGA (B) and TCGA (D) datasets stratified by IDH status. * $P<0.05$, ** $P < 0.01$ and **** $P < 0.0001$.

Figure S2. The expression of eIF3d/e/f/h/l in IDH-mutant and IDH-wildtype glioma patients with different gender.

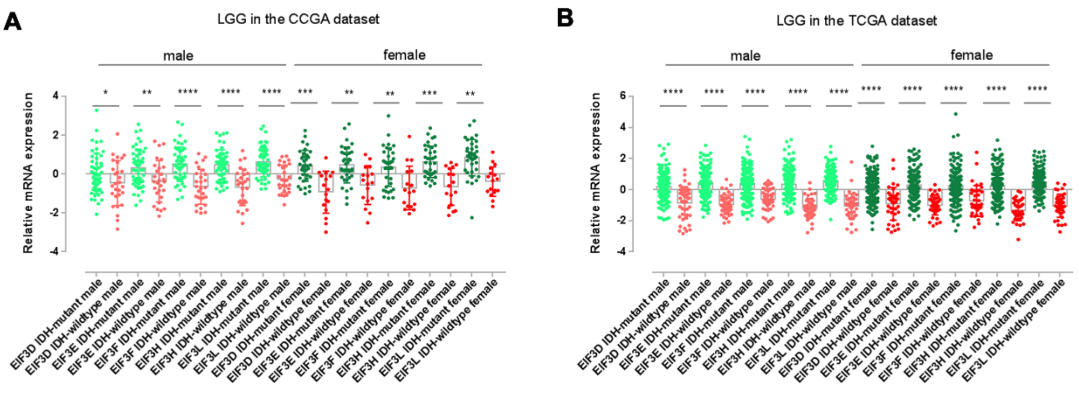

(A-B) Quantification data shows the expression levels of eIF3d/e/f/h/l in gliomas from the CGGA (A) and TCGA (B) datasets stratified by IDH status. * P<0.05, ** P < 0.01, ** P < 0.001 and **** P < 0.0001.

Figure S3. Relation between eIF3i expression and 1p/19q codeletion status in IDH-mutant LGG

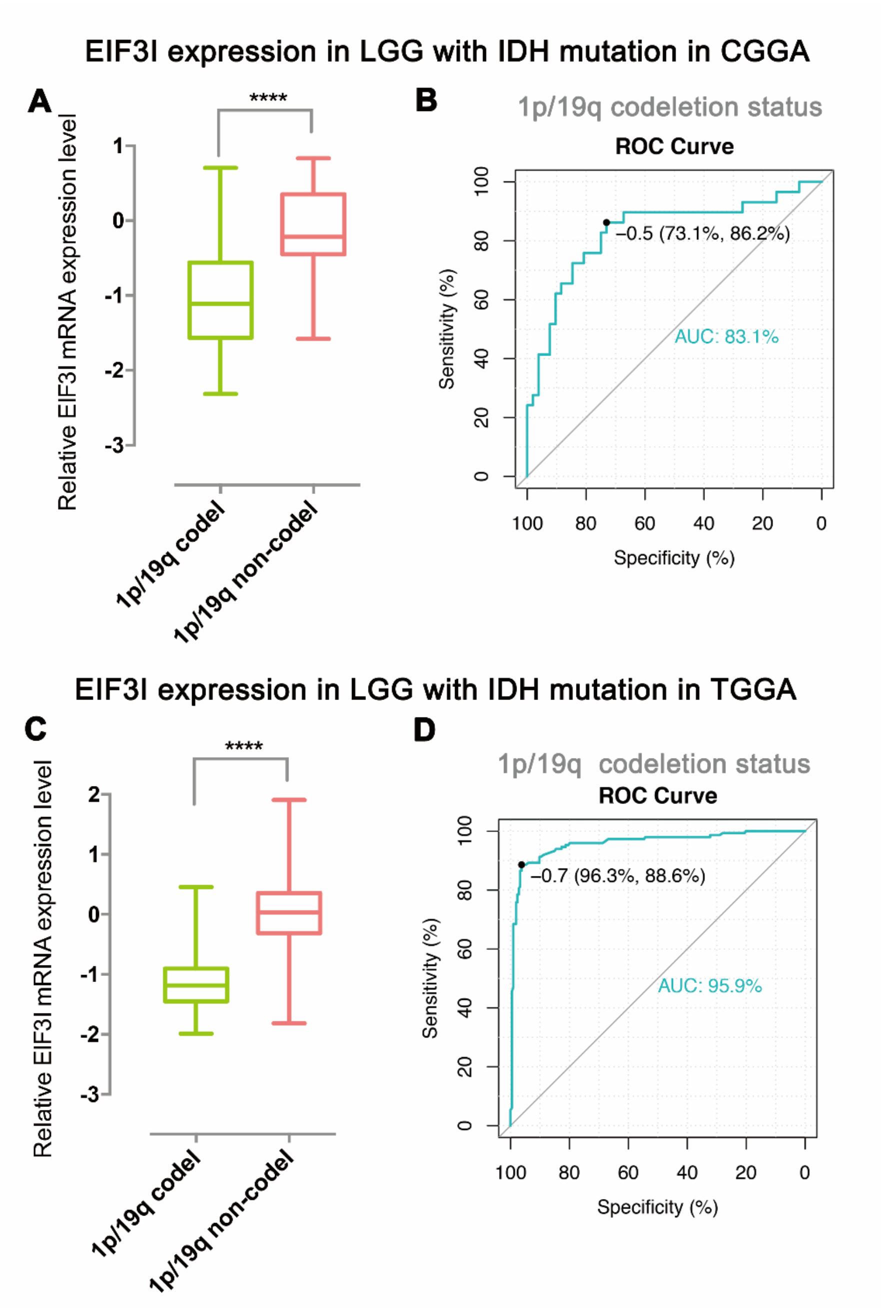

(A and C) Distribution of eIF3i in IDH mutant LGG stratified 1p/19q codeletion status from the CGGA (A) and TCGA (C) datasets. **(B and D)** ROC cures showed the predictive efficiency of the eIF3i expression on the 1p/19q codeletion status of IDH mutant LGG in CGGA (B) and TCGA (D) datasets.

Figure S4. The prognostic value of eIF3i in total glioma, LGG and GBM, respectively.

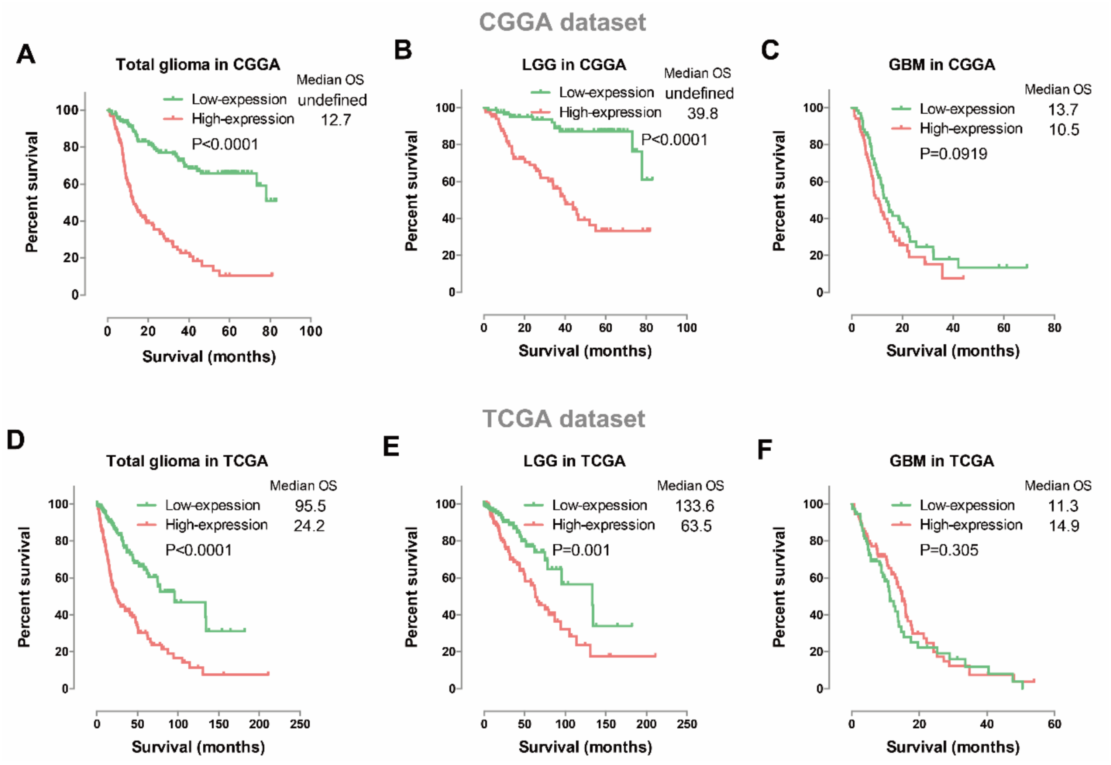

(A-F) Kaplan–Meier overall survival curves for patients stratified by the respective median expression of eIF3i in the CGGA (A-C) and TCGA (D-F) datasets with total gliomas (A and D), LGG (B and E), and GBM (C and F), respectively.

Figure S5. The prognostic value of eIF3i in stratified GBM.

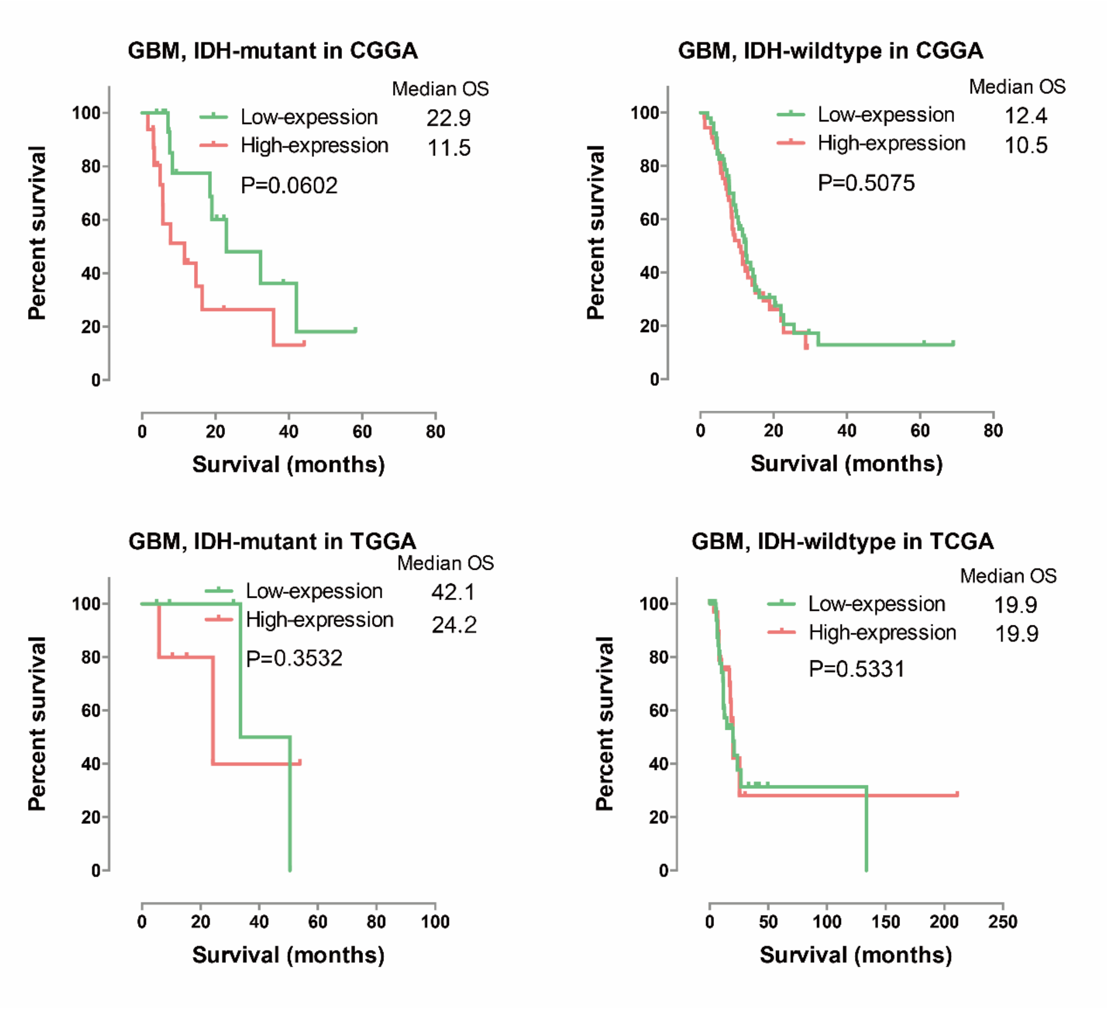

Kaplan–Meier overall survival curves for patients stratified by the respective median expression of eIF3i in the CGGA and TCGA datasets with IDH-mutant GBM and IDH-wildtype IDH, respectively.
